# Using Collaborative Partnerships to Engage Firefighters in Rural Communities

**DOI:** 10.3390/ijerph19042009

**Published:** 2022-02-11

**Authors:** Ritchie Taylor, Gretchen Macy, Jooyeon Hwang, Vijay Golla, Charles Cann, Edrisa Sanyang

**Affiliations:** 1 Center for Environmental and Workplace Health, Department of Public Health, College of Health and Human Services, Western Kentucky University, Bowling Green, KY 42104, USA; ritchie.taylor@wku.edu (R.T.); charles.cann@wku.edu (C.C.); edrisa.sanyang@wku.edu (E.S.); 2 Department of Occupational and Environmental Health, Hudson College of Public Health, University of Oklahoma Health Sciences Center, Oklahoma City, OK 73104, USA; jooyeon-hwang@ouhsc.edu; 3 Department of Life Sciences, Texas A&M University-San Antonio, San Antonio, TX 78224, USA; vijay.golla@tamusa.edu

**Keywords:** occupational health, firefighter health, occupational safety and health, community-based participatory research (CBPR), exposure assessment, airborne contaminants, carcinogens

## Abstract

The purpose of this study was to utilize community-based participatory research (CBPR) methods to identify behaviors that may increase the exposure rates of firefighters to carcinogens and other occupational hazards. Key informant interviews and focus groups were conducted as part of a larger study that included exposure assessments at fire stations, in fire engines, and in personal vehicles. A purposive sample of five fire chiefs and leaders of the firefighter association was chosen, and these individuals were selected to participate in interviews. Unstructured interviews explored issues related to firefighter exposures and occupational hazards. Three focus groups were conducted over a three-month period. Following the focus groups, survey questions emphasizing the following three aspects of firefighting were developed: exposure to airborne smoke contaminants during fire suppression, accumulated exposure on turnout gear after fire suppression, and exposure to airborne contaminants at the fire departments. The use of community-based participatory research can be very beneficial, especially when conducting research with a group that may be hard to reach or have misgivings regarding researchers. By utilizing this approach in the current study, researchers were able to partner with a community that may be hard to reach and gain buy-in from community leadership.

## 1. Research Approach

According to the National Fire Protection Association (NFPA), there were approximately 1,115,000 firefighters in the United States (U.S.) in 2018. Among U.S. firefighters, 370,000 (33%) were career firefighters, while 745,000 (67%) were volunteer firefighters. The majority of career firefighters (67%) work in jurisdictions that protect 25,000 or more people. Almost half (49%) of volunteer firefighters serve in departments that are rural and small, protecting fewer than 25,000 people. Of the 29,705 fire departments in the U.S., approximately 10% are made up of strictly career firefighters. Conversely, 19,122 (64%) are composed entirely of volunteer firefighters [1]. Departments protecting more than 25,000 people were more likely to be served by career firefighters (75–100%), while departments serving fewer than 25,000 people were more likely to be serviced by volunteer firefighters (49–97%). Additionally, departments serving less than 25,000 people were more likely to have younger firefighters under the age of 30, while departments serving fewer than 2500 people were more likely to have firefighters over the age of 50, compared to departments serving larger jurisdictions [1]. Of the 683 Kentucky fire stations registered through the U.S. Fire Administration, 76% are registered as volunteer only, 14% are registered as mostly volunteer, 4% are registered as mostly career, and 6% are registered as career only. Kentucky currently ranks 17th in the United States according to the percentage of volunteer fire departments, with 90.6% of all registered departments classified as all or mostly volunteer [2]. Among Kentucky’s approximately 21,000 firefighters, 17,000 are volunteers. According to the Kentucky Fire Commission’s report, there are currently 776 fire departments in the commonwealth, though some are not registered with the U.S. Fire Administration. Of the 776 departments, 621 are volunteer departments [3].

Increased exposures to asbestos, diesel particulates, polycyclic aromatic hydrocarbons (PAHs), volatile organic compounds (VOCs), and hydrogen cyanide (HCN) among firefighters places them at an increased risk of adverse health outcomes [4,5,6,7,8]. Research has shown that occupational exposures increase the risk of on-duty heart attack, cardiovascular disease, respiratory illness, and injury [5,6]. Firefighters’ exposure to a variety of toxic substances may lead to the development of certain cancers, such as myeloma and cancers of the esophagus, intestine, kidneys, colon, and lungs [4,5,6,7,8]. Findings from the National Institute for Occupational Safety and Health [6] studies have shown that firefighters have a greater prevalence of cancer diagnoses and cancer-related deaths than the general public. Improper decontamination and storage procedures result in the accumulation of carcinogens on firefighter turnout gear, including the helmet, hood, coat, pants, and boots. Additionally, transportation, including personal vehicles, may be contaminated while transporting gear to and from response events. Field observations indicate that volunteer firefighters regularly store turnout gears in their personal vehicles [9]. Toxic substances on the gear may then be transferred to the firefighters’ skin. Additionally, studies indicate that off-gassing from turnout gear leads to additional exposures to these substances, particularly VOCs and HCN, creating a toxic environment surrounding the place in which the turnout gear is stored [10].

In order to reduce firefighter exposures, three factors may be considered: (1) Where is the greatest magnitude of exposure? (2) What behaviors increase the risk of exposure? (3) What factors impact exposure? The goal of the overall study was to explore each of these factors. While there have been large scale studies conducted with career firefighters living in urban areas, there have been few studies conducted with volunteer firefighters, especially in rural areas. Reasons for the limited number of studies are that volunteer firefighters in rural areas have different full-time jobs, are more difficult to engage due to location, and may be less trusting of researchers due to rural culture [11,12]. Tanner et al. (2013) found that rural populations were less likely to take part in research compared to the general public [11]. Similarly, Miyamoto et al. found that recruiting rural participants for behavioral health interventions was challenging. However, participation was increased through the use of trusted site coordinators. This shows the importance of building a long-term collaborative relationship with rural populations that may be sustained over time as trust is built [12].

Community-based participatory research (CBPR) focuses on foundational trust and sustainability. Community-based participatory research involves including the public or a specified community in the exploration of an occupational health problem. CBPR may be defined as “a partnership approach to research that equitably involves community members, organizational representatives, and academic researchers in all aspects of the research process. It enables all partners to contribute their expertise, with shared responsibility and ownership; it enhances the understanding of a given phenomenon; and, it integrates the knowledge gained with action to improve the health and well-being of community members, such as through interventions and policy change” [13,14]. While implementing a CBPR framework, a partnership is formed between a research institution and the community—in this case, firefighters—to initiate, plan, and conduct research to address occupational safety and health issues [13,14,15,16,17]. This collaborative approach involves community members, organizational representatives, and researchers throughout the research process [14]. The use of community-based research allows all included individuals to contribute their unique strengths, as well as share responsibilities [14].

Israel et al. [14] identified the key factors and benefits of community-based research: (1) recognizing the community as a unit of identity, (2) building on strengths and resources within the community, (3) facilitating collaborative partnerships, (4) integrating knowledge and action for the mutual benefit of all individuals involved, (5) promoting a co-learning and empowering process, (6) involving a cyclical and iterative process, (7) addressing health from various perspectives, and (8) disseminating the knowledge and findings gained to all individuals involved [14]. The first principle of CBPR acknowledges that each community has an identity with shared norms, values, goals, and interests. Research partnerships attempt to enhance the sense of community through collaborative efforts. Building on community strengths and resources is another principle of CBPR. Through this framework, social networks, skills, and sociocultural structures, such as community and faith-based organizations, are utilized to synergize improvements in quality of life. At its core, CBPR is built on collaboration, and promotes the reciprocal transfer of increased capacity, skills, and knowledge [13]. These collaborations have the goal of providing mutual benefit for all participants, while attempting to identify and address community concerns.

The next principle of CBPR involves the equitable and collaborative partnerships that involve empowerment and power sharing. CBPR calls for the sharing of control over the process and calls for open communication. Researchers understand the potential for inequitable power in the research process, and make intentional efforts to share the resources, decision making, and information at each stage of the process. An additional principle of CBPR is the focus on long-term sustainability. Relationships and collaborations are the foundation of CBPR, and the expectation is that these partnerships last well beyond one research project, and instead lead to a sustainable relationship spanning many years. An ecological perspective is also applied to CBPR, as emphasis is placed on examining multiple determinants of health throughout the process. Finally, the last principle of CBPR includes the dissemination of research findings to the broader community, ensuring that all partners are aware of results and are able to use the knowledge to improve the health of the community [13,14].

**Figure 1:**
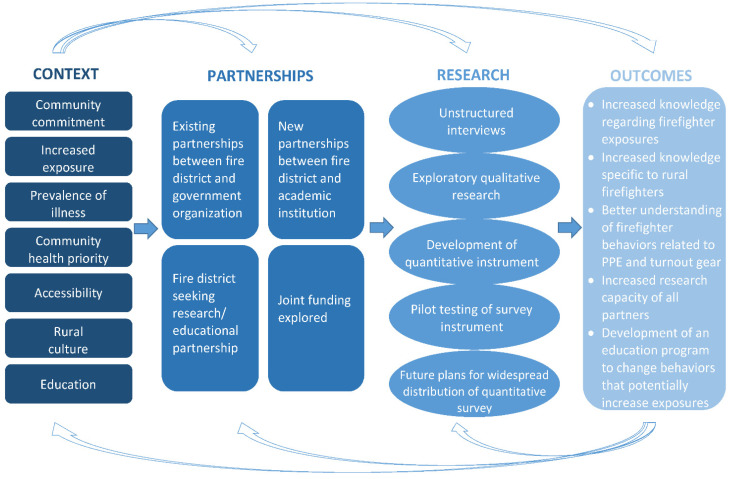
Conceptual Model of CBPR with Rural Firefighters. Adapted from Wallerstein et al. (2010) [17].

Delisle et al. utilized the components of CBPR to implement a successful academic partnership with firefighters to promote physical activity and reduce cardiovascular disease [18]. Another study [19] examined the collaboration between public environmental health and the emergency preparedness and response team. Gamboa-Maldonado et al. found that “training in participatory methods is needed to protect knowledge and emergency management” while strengthening the community and more efficiently carrying out emergency response [19]. Based on these and other findings, CBPR was the chosen research approach for the research plan because firefighters are disproportionally affected by certain types of cancer, and there was a desire to develop a community collaboration that would address these health issues from various perspectives. Additionally, volunteer and rural firefighters have been shown to be more difficult to engage in research, as evidenced by the limited studies in the literature. This rationale was used to determine the need for a research approach that included substantial community involvement [13,14,15,16,17,18,19]. The conceptual model for the implementation of the CBPR approach to this research study is shown in Figure 1.

The purpose of this study was to utilize CBPR methods to identify behaviors that may increase firefighters’ exposure rates to carcinogens and other occupational hazards. The methodology allowed for the elicitation of information from firefighters to develop a survey instrument that may be disseminated to a larger population. This was achieved by developing a stakeholder group, contacting gatekeepers to act as key informants, conducting focus groups, and developing a quantitative survey instrument.

## 2. Activities

The academic institution and firefighter association described in this study have partnered for four years on research with the goal of developing a survey instrument to assess behaviors related to turnout gear. The information obtained through the assessment may lead to educational programs and policy initiatives in rural firefighter communities. An environmental and workplace health research center provided the infrastructure for academic units, government organizations, private industry, and the community to collaborate on efforts to improve the safety and health of workers. The center provided faculty with expertise in exposure science and qualitative data collection, and supported the administration of grant funding to carry out the project. Based on community identified needs and National Institutes for Occupational Safety and Health (NIOSH) priority research areas, a stakeholder group of firefighter community members, academic researchers, government officials, and firefighter organizational leaders was established to investigate and monitor the unique exposures of volunteer and career firefighters in a rural state. The goals of the CBPR approach include developing educational programs and policy implementation to reduce the exposure of firefighters to occupational hazards, specifically carcinogens (See Figure 1) [17].

This study was approved by the University’s Institutional Review Board (IRB code No.: 16-446). Informed consent was obtained from all firefighters prior to participation. Key informant interviews and focus groups were conducted as part of a larger study that included exposure assessments at fire stations [20], in fire engines, and in personal vehicles [21]. Researchers partnered with a rural firefighter association that included 70 independent fire stations, both volunteer and career, over an eight-county area in a rural state. One of the researchers had been working with the community for ten years, providing air quality expertise as a government official. The close relationship between this researcher and the community was paramount in gaining trust to move forward with the CBPR initiative.

This association includes 70 fire departments in eight counties in a rural location that represents 10% of the fire departments in the state A purposive sample of five fire chiefs and leaders of the firefighter association were selected to participate in interviews [22,23]. Brief, unstructured interviews explored issues related to firefighter exposures and occupational hazards. Based on these interviews, exposures at the fire scene and at the fire station, as well as other behaviors related to the use of Personal Protective Equipment (PPE) and turnout gear, were identified as areas needing further exploration by the CBPR group. These key areas were utilized in the development of a script for focus group discussions.

Next, three focus groups were conducted over a three-month period. Eight members that were firefighter leaders attended each of the first two focus groups from the fire departments in the firefighter association. The goals of the research and the procedures for the discussion were explained by the facilitator prior to the start of the focus group. The discussion focused on exposure to air contaminants when fighting fires. The topics discussed were as follows: the benefits of being a firefighter, the major barriers of performing the duties of a firefighter, barriers to fire suppression, barriers to working in the fire station, barriers to cleaning and properly maintaining turnout gear, ways to reduce exposure to air contaminants while on the job, ways to reduce smoke contamination when suppressing a fire, ways to reduce air contamination while at the station, ways to reduce the accumulation of contaminants on turnout gear, major concerns about job duties during fire suppression, procedures for monitoring and measuring the exposures in the air, routines related to turnout gear, the storage of gear, the cleaning of gear, potential procedural changes related to the storage and cleaning of gear, additional concerns regarding air contaminates and occupational exposures during fire suppression and at the fire station, and additional comments or concerns regarding accumulated contaminants on turnout gear and the maintenance of one’s gear. Additional information, including gender, years of experience, main job tasks, etc., was collected (see Supplemental Materials). Focus groups are particularly useful when existing knowledge of a subject is inadequate and the subject under investigation is complex [24,25]. As compensation for their time and effort, a meal and a USD 25 gift card were provided to all focus group participants. Each meeting was approximately 90 min. The discussions in all focus group sessions were strictly confidential, and no personal information was collected. The information obtained from focus groups was recorded with permission by a scribe. Each de-identified transcript was coded into themes by the research group to develop an initial list of topics related to exposures. These themes were then used to develop a quantitative instrument aimed at gaining insight into firefighter behaviors and exposure risks [22].

Based on information obtained from the facilitated focus groups, a survey instrument was developed that focused on three aspects of firefighting: exposure to airborne smoke contaminants during fire suppression, accumulated exposure on turnout gear after fire suppression, and exposure to airborne contaminants at the fire department. After the survey was developed, an additional focus group was held where the same leaders were interviewed to provide feedback and face validity for the first version of the survey instrument. This discussion was focused on survey instrument design, logistical issues, such as the administration of the survey, and the survey instrument distribution on the survey day. Following the second meeting with the fire leadership, the respective firefighter leaders in the first discussions sent an invitation email to the firefighters of their local districts. Eleven voluntary participants were randomly selected from the respondents. In this group discussion, the revised survey instrument was discussed to solicit the participants’ feedback on fire smoke and occupational exposures. The participants held frontline jobs, such as firefighters and instructors. Thus, the discussion was intended to obtain the firefighters’ perceptions of the exposures they encountered, the obstacles they faced, and the support they received on the job. After this interview, the third version of the survey instrument was finalized for distribution to the firefighter cohort group.

Following a thematic analysis of the focus groups, areas of concern were determined to be air borne exposure at the fire scene, the accumulation of contaminants on turnout gear, and airborne exposures at the fire station. Primary concerns were the increased rates of cancer among the firefighter population and the potential impact of occupational exposures on health. Based on these findings, survey questions emphasizing the following three aspects of firefighting were developed: exposure to airborne smoke contaminants during fire suppression, accumulated exposure on turnout gear after fire suppression, and exposure to airborne contaminants at the fire departments. Since research [4,5,6,7,8] indicates that increased occupational exposures among firefighters lead to an increased risk for multiple cancers, and because the firefighters indicated that cancer was the major concern during the focus groups, the researchers focused on cancer as the primary adverse health outcome in the survey instrument. Following the development of the survey, the participants from focus groups were reconvened to review the survey instrument. During the focused discussion, participants provided feedback to increase the face validity of the original instrument. This discussion focused on the content and clarity of the survey instrument, logistics, such as the mode of administration of the survey, and potential distribution channels for the survey. The findings of these discussions led to minor changes in the terminology used in survey questions, the change to a fully pen-and-paper survey as opposed to the planned online survey, and the addition of questions related to the perception of health risks due to job-related exposures.

After feedback was obtained, the original survey instrument was revised based on participant comments. The revised survey was then distributed to participants in an additional group for review. Gatekeepers in the firefighter association sent an invitation e-mail to the firefighters in the association. Eleven volunteer participants were randomly selected from the respondents. During this group, the revised survey instrument was discussed to solicit the participants’ feedback regarding fire smoke and occupational exposures. The participants held frontline jobs, such as firefighters and instructors. Thus, the discussion aimed to obtain the firefighters’ perceptions of the exposures they encountered, the obstacles they faced, and the support they received on the job. After this interview, the third version of the survey instrument was finalized for distribution for a pilot test. Due to the majority of both the leadership and frontline firefighters raising concerns about the health risks associated with job-related exposures, additional questions based on the health belief model were added to the final version of the survey. These questions operationalized the constructs of self-efficacy, cues to action, perceived threat (perceived severity and perceived susceptibility), perceived benefits, and perceived barriers to the proper cleaning, storage, and maintenance of turnout gear.

Based on the information obtained from the second and third survey reviews, a 46-item questionnaire was developed. The questionnaire will collect information on demographics, exposures at the fire station, fire scene related exposures, and behaviors related to the use of PPE. The information gathered from this survey will be utilized to develop an educational program and inform policy related to PPE use and storage, and the replacement of turnout gear, to reduce exposures among volunteer, rural firefighters.

## 3. Conclusions

Issues of exposure to airborne smoke contaminants during fire suppression, accumulated exposure on turnout gear after fire suppression, and exposure to airborne contaminants at the fire departments were explored during the academic–community health partnership meetings. Firefighter participants in the study were most concerned about cancer. This finding is consistent with other studies, which have identified that the primary health concern among firefighter is the development of cancer [26]. By utilizing the CBPR approach, a survey was developed to further explore behaviors that are relevant to increased exposures, written with terminology that firefighters utilize. Additionally, the partnership allowed firefighters to be accessible and involved in each step of the process [21].

The strength of this project was that it led to community engagement and empowerment. Through participation in the academic–community partnership, community members became more likely to seek information and engage in problem solving to change the identified areas of exposures related to behavior change. The academic institution become infused into the firefighter activities, including having an agenda item in the regional firefighter association bi-monthly and annual meetings to not only provide research updates, but also hear first-hand information about research issues that matter to the rural firefighters. It was also anecdotally shown that the study increased the awareness of behaviors leading to increased exposure and knowledge of how to reduce exposures, especially among the fire chiefs and administrators. This study was part of a larger research collaboration between an academic institution and a regional firefighter organization. The larger project applies the principles of CBPR in the following ways: the project recognizes firefighters as a community with their own set of knowledge, values, and skills; the collaboration builds on the strengths of infrastructure, loyalty, and procedural expertise; the project facilitates collaboration by fostering relationships among firefighters, researchers, and the larger first-responder community; there is the transfer of knowledge that is mutually beneficial for all, as firefighters have helped with the development of survey instruments and training, and the researchers have helped to identify ways to reduce exposures to firefighters; all participants are empowered as skill and capacity building is taking place to help improve the health of the community; the process is on-going, as the collaboration started over four years ago, and the project has expanded to address other challenges, such as COVID-19 exposures; the partnership examines multiple determinants of health from the intrapersonal and interpersonal levels to the policy level; information is consistently shared among the group through individual channels and personal communication to professional presentations at yearly fire schools.

The challenges of conducting CBPR may include preconceived stereotypes of both the researchers and community members [25], though this did not seem to be a barrier during the key informant interviews. It is possible that participants were guarded about what they chose to share during the focus groups; however, this was not disclosed at any point. Participant misgivings and stereotyping were potential issues when developing the survey, as rural firefighters were less likely to want to participate in the validation of the survey. Additionally, rural Americans are more likely to be poor, uninsured, obese, and use tobacco. Rural Americans are also less likely to have access to healthcare, with only 9% of physicians in the United States practicing in rural settings [27,28]. The combination of increased exposures, the increased likelihood of comorbidities, and reduced access to healthcare could all lead to poorer outcomes for rural firefighters. This further shows the need for partnerships where all parties are equally represented at each stage of research, from problem identification to decision making and policy change.

## 4. Implications/Recommendations

The use of CBPR methods can be very beneficial, especially when conducting research with a group that may be hard to reach or less receptive to participating in research. Unique barriers have been identified to recruiting participants in rural areas, such as culture, knowledge, and attitudes [27]. CBPR methods are useful when little is known about the topic of inquiry, in this case, exposure to hazards associated with rural firefighters and, specifically, volunteer firefighters working in rural areas. Studies have shown that rural communities are more likely to take part in research if they feel that it is relevant to their needs, there is two-way communication, and they are empowered [29]. By utilizing this approach in the current study, researchers were able to partner with a community that may be hard to reach, and gain buy-in from community leadership through a multi-year, multi-project approach. By working with gatekeepers and leaders, researchers were able to access all membership levels within the firefighter community, from leadership to part-time volunteers. This access provided information regarding the exposures volunteer firefighters may have while on the job, and how certain behaviors may lead to additional exposures after leaving the fire scene.

The information gained through this CBPR project is invaluable for the development of interventions to educate firefighters about behaviors that may place them, and their families, at increased risk of illness and disease, as well as inform policy to reduce exposures. Additionally, this work can serve as a guide to other researchers aiming to engage communities in rural locations. By applying the components of CBPR, building rapport, and establishing mutually beneficial relationships, meaningful collaborations between academic institutions and rural populations may be successful.

## Data Availability

Data are available upon request.

## Supplementary Materials

The following supporting information can be downloaded at: https://www.mdpi.com/article/10.3390/ijerph19042009/s1.
