# Supplementary material for: Using Collaborative Partnerships to Engage Firefighters in Rural Communities
Source: Int J Environ Res Public Health. 2022 Feb 11;19(4):2009. doi: 10.3390/ijerph19042009 (PMC8871703; doi:10.3390/ijerph19042009)
Supplement: Supplementary file 1 [file ijerph-19-02009-s001.zip › ijerph-1551309-supplementary.pdf]

Supplementary Materials: Questions for the focus groups:

(1) Please discuss some of the benefits of your job as a firefighter.
(2) Please discuss some of the major barriers you face when trying to perform your job.
- o What barriers do you face when suppressing a fire?
- o What barriers do you face when working in the fire station?
- o What barriers do you face when cleaning and maintaining your gear?

(3) What would reduce your exposure to air contaminants when performing your job?
- o What would reduce the potential of fire smoke contamination when suppressing a fire?
- o What would reduce the potential of air contamination while at the station?
- o What would reduce the potential accumulation of contaminants on your turnout gear?

(4) What are your major concerns about your job duties during fire suppression?
(5) How does the fire department monitor and measure the exposures in the air?
(6) Briefly discuss your routine for turnout gear, including where you store your gear and how you have it cleaned.
- o What could be changed to make meeting the standard for turnout gear more feasible?

(7) Please discuss any additional comments or concerns you have regarding air contaminates and occupational exposures during fire suppression and at the fire station.
(8) Please discuss any additional comments or concerns you have regarding accumulated contaminants on your turnout gear and the maintenance of your gear.

In addition, we also asked a basic information including gender, years of experience, main job tasks, etc.
